# Chromosome-Scale Genome Assembly for Chinese Sour Jujube and Insights Into Its Genome Evolution and Domestication Signature

**DOI:** 10.3389/fpls.2021.773090

**Published:** 2021-11-24

**Authors:** Lian-Ying Shen, Hang Luo, Xiao-Ling Wang, Xue-Meng Wang, Xiao-Jing Qiu, Hui Liu, Shan-Shan Zhou, Kai-Hua Jia, Shuai Nie, Yu-Tao Bao, Ren-Gang Zhang, Quan-Zheng Yun, Ying-Hui Chai, Jin-Ying Lu, Yu Li, Shu-Wei Zhao, Jian-Feng Mao, Shan-Gang Jia, Yong-Min Mao

**Affiliations:** ^1^Research Center of Chinese Jujube, Hebei Agricultural University, Baoding, China; ^2^Beijing Advanced Innovation Center for Tree Breeding by Molecular Design, National Engineering Laboratory for Tree Breeding, College of Biological Sciences and Technology, Beijing Forestry University, Beijing, China; ^3^College of Grassland Science and Technology, China Agricultural University, Beijing, China; ^4^Beijing Ori-Gene Science and Technology Co., Ltd., Beijing, China; ^5^Shenzhou Space Biotechnology Group, China Academy of Space Technology (CAST), Beijing, China; ^6^Institute of Geographic Sciences and Natural Resources Research, Chinese Academy of Sciences (CAS), Beijing, China; ^7^Hebei Hemuliyuan Agricultural Science and Technology Co. Ltd., Baoding, China

**Keywords:** sour jujube, genome assembly, stress response, fruits, selective sweep

## Abstract

Sour or wild jujube fruits and dried seeds are popular food all over the world. In this study, we reported a high-quality genome assembly of sour jujube (*Ziziphus jujuba* Mill. var. *spinosa*), with a size of 406 Mbp and scaffold N50 of 30.3 Mbp, which experienced only γ hexaploidization event, without recent genome duplication. Population structure analysis identified four jujube subgroups (two domesticated ones, i.e., D1 in West China and D2 in East/SouthEast China, semi-wild, and wild), which underwent an evolutionary history of a significant decline of effective population size during the Last Glacial Period. The respective selection signatures of three subgroups were discovered, such as strong peaks on chromosomes #3 in D1, #1 in D2, and #4 in wild. Genes under the most significant selection on chromosomes #4 in wild were confirmed to be involved in fruit variations among jujube accessions, in transcriptomic analysis. Our study offered novel insights into the jujube population structure and domestication and provided valuable genomic resources for jujube improvement in stress response and fruit flavor in the future.

## Introduction

Chinese jujube, which is known as sour jujube called “Suanzao,” acid jujube, or wild jujube, is a tree species (*Rhamnaceae*: Dicotyledoneae) with a high tolerance of drought, cold, waterlogging, and barren (Qu and Wang, 1993). As the native species of jujube, Chinese jujube (*Ziziphus jujuba* Mill. var. *spinosa*) is usually produced as a root stock for jujube. Sour jujube is distributed all over the world, with a good adaption to arid regions and tolerance of a range of climate conditions (Johnston, 1963; Outlaw et al., 2002). There are many old jujube trees, having been lived for hundreds of years, in northern China, such as Shanxi and Hebei. It is significant native vegetation in the Taihang and Yanshan mountains in China.

Sour jujube is well known for the nutritional value of the fruit, the medicinal importance of the seeds. Although its tastes are not as sweet as the other jujube, its fruits and dried seeds (i.e., *Zizyphi Spinosi Semen*, SZS) have long been used in traditional Asian medicine. Many active compounds, e.g., polyphenols, flavonols, polysaccharides, and anthocyanins, in the fruit possess biological and antioxidant activities (Ji et al., 2020). More than 130 compounds enriched in the seeds, including saponins, flavonoids, alkaloids, and fatty acids, have been isolated and identified from sour jujube seeds (Cheng et al., 2000). Modern pharmacological research showed that sour jujube seeds had a wide range of pharmacological effects, such as sedative and tranquilizing, anti-aging, anti-anxiety, anti-depression, anti-tumor, and myocardial protection (Sun et al., 2011). Eight flavonoid compounds were found, including swertish, puerarin, isospinosin, flavonols, and flavan-3-ols (Ji et al., 2017; Lin et al., 2018). Polysaccharide extracts showed certain antioxidant activities, including scavenging ABTs radical, superoxide radical, hydroxyl radical, and ferrous ion *in vitro*, and inhibiting reactive oxygen species (ROS) accumulation (Lin et al., 2018).

Sour jujube is one of the wild fruit trees with great ecological and economic value in greening barren hills and controlling deserts (Liu and Zhao, 2009; Zhang et al., 2015). For example, in the mid-1990s, sour jujube made a great contribution to the large-scale construction of fruit production bases for desert management in Xinjiang Province, China (Wang and Sun, 1986; Liu M. et al., 2020). After planting sour jujube seeds, the length of the root system was dozens of times bigger than that of the aboveground seedlings, which is key to making use of limited water deep in the desert (Wang et al., 2016). If grafting jujube in the next spring after seeding, it could be fruitful in the same autumn (Shi et al., 2016). As a result, more than 53 thousand ha of jujube forest was successfully constructed in Xinjiang (Song et al., 2019).

Although sour jujube has importance in breeding and studies of plants responding to stress, the information of its genome and genetic diversity is still lacking, which limits the utilization of genetic information. Recently, the genomic study on *Z. jujuba* made a breakthrough. The genomes of *Z. jujuba* Mill. cultivars “Junzao” and “Dongzao” (2n = 24), differing from *Z. jujuba* Mill. var. *spinosa* (i.e., Suanzao), was assembled with the genome size of around 443 Mbp/360 Mbp and scaffold N50 of only ∼301 kbp/755 kbp (Liu et al., 2014; Huang et al., 2016), based on the genetic map, BAC sequencing, and Illumina sequencing. In this study, a high-quality genome assembly, scaffold N50 of 30.3 Mbp, for *Z. jujuba* Mill. var. *spinosa* was present, by using a combination of PacBio single-molecule real-time (SMRT) sequencing, Illumina HiSeq short-read sequencing, and high-throughput chromatin conformation capture (Hi-C) for chromosome-level assembly. Based on the assembled genome and resequencing, the population structure was analyzed, and 109 jujube accessions were classified into four subgroups, i.e., D1, D2, semi-wild and wild. We demonstrated that the four subgroups experienced different histories of genome evolution and selection signatures were discovered on different chromosomes. Selection signatures on chromosome #10 enriched with stress-responding genes were discovered in all three groups, and the transcriptomic analysis confirmed their functions in fruit variations among jujube accessions. The genome assembly and the results in this study provide a valuable and excellent reference for comparative genomic analysis with the available jujube genomes, and genomic dissection of genetic diversity of wild jujube, which will benefit the molecular breeding of sour jujube in the future and studies of the response of plants to stresses and fruit flavor improvements.

## Materials and Methods

### Plant Material

**FIGURE 1:**
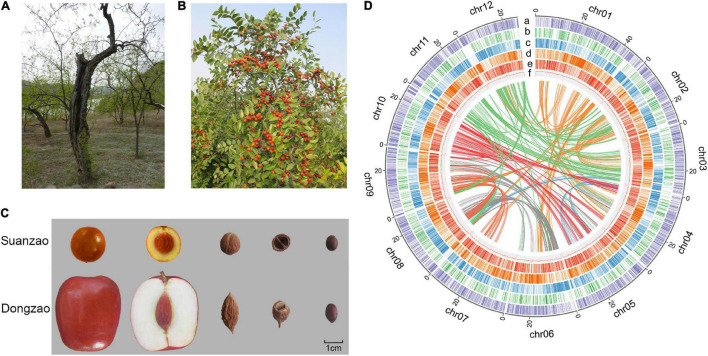
Fruit and dried seed of *Z. jujuba* Mill. var. *spinosa* (Suanzao or sour jujube). **(A)** The Suanzao tree that was used for sample collection in the genome sequencing. **(B)** Mature Suanzao fruits on the tree. **(C)** Comparison of Suanzao and Dongzao (*Z. jujuba* Mill.) for the fruit and seed. **(D)** Circular plot of the pseudomolecules in *Z. jujuba* Mill. var. *spinosa*. The coordinates of 12 pseudomolecules are shown in Mbp. The layers are placed from the outside to the inside, a: density of Copia long terminal repeat-retrotransposons (LTR-RTs); b: density of LINE LTR-RTs; c: density of genes; d: density of Gypsy LTR-RTs; e: pseudogene density; and f: GC content. Central lines connect syntenic blocks across pseudomolecules, and colors were randomly selected to represent different connections.

A sour jujube tree grafted on a millennium-old rootstock, which has germinated several new branches and blossomed normally in recent years (Figure 1A), was selected as the sequencing material. This ancient tree is located in the jujube forest close to the Yellow River on Linxian County, Shanxi Province, China (N37°59′ latitude, E110°31′ longitude, 717 m elevation). Fresh leaves, flowers, young fruits, and stems were collected for transcriptome assembly from May to July 2017. Samples were immediately transported on dry ice to the lab for sequencing.

### Genome Sequencing

High molecular weight genomic DNA was extracted from the leaf tissues using QIAGEN DNeasy Plant Max Kit (Qiagen, Valencia, CA, United States), and purified using the Mo-Bio PowerClean Pro DNA Clean-Up (Qiagen, Hilden, Germany). DNA quality was evaluated *via* standard agarose gel electrophoresis and Thermo Fisher Scientific Qubit Fluorometry (Thermo Fisher Waltham, MA, United States). High-quality DNA was sheared to ∼20 Kbp targeted size, based on the standard protocols of Pacific Biosciences company, which was followed by being enzymatically repaired (damage repair and end repair), adaptor ligation, size selection for SMRTbell template libraries construction, and sequencing on the PacBio Sequel platforms. For Illumina sequencing, high-quality DNA was further purified by being incubated with Proteinase K and RNaseA at 25° for 30 min. Purified DNA was sheared, end-repaired, adenylation tailed, universal adapter-ligated, and indexed, for the construction of barcoded libraries, according to the protocol of the manufacturer. The whole-genome library was sequenced on the Illumina HiSeq platform.

The Hi-C process was performed following the protocol of the manufacturer. Young leaf tissue was fixed in 1% formaldehyde solution and nuclei were extracted, followed by being digested with *Hin*dIII. The sticky ends from *Hin*dIII digestion were filled in with biotinylated nucleotides and ligase. Finally, DNA was then sheared to ∼350 bp and a *situ* Hi-C library was constructed. The library was sequenced on Illumina HiSeq 2500 platform under paired-end 150 bp mode.

Ribonucleic acid was isolated from stem, leaf, flower, and fruit using the NEBNext Poly (A) mRNA Magnetic Isolation Module (New England Biolabs, United States), and then RNA quality was evaluated on Agilent 2100 BioAnalyzer (Agilent Technologies, Palo Alto, CA, United States). Four sequencing libraries were prepared for Illumina sequencing using the NEBNext Ultra RNA Library Prep Kit (New England Biolabs, United States). All the libraries were sequenced on an Illumina HiSeq 2000/2500 machine with a 150/100 bp pair-end sequencing strategy.

### Genome Survey

The software tool, Jellyfish, was used to count the occurrence of k-mer based on Illumina short reads before genome assembly (Marçais and Kingsford, 2011). A total of 24,069,245,190 k-mers (K = 17) were identified, and the peak of k-mer depth was 60 (Supplementary Figure 1 and Supplementary Table 2). Results of Jellyfish were input into the GCE version 1 to estimate the genome size, repeat content, and the heterozygosity of Suanzao (Liu et al., 2013). Finally, the genome size was calculated to be ∼ 413 Mbp.

### Genome Primary Assembly

There were three steps of genome assembly, primary assembly, Hi-C scaffolding, and polishing. PacBio long reads, which were corrected by Canu version 1.6 (Koren et al., 2017), were used for primary assembly. Assembly version.1 was generated by SMARTdenovo version 1 (Liu H. et al., 2020), assembly version.2 by Wtdbg version 1.2.8,^1^ assembly version.3 by SMARTdenovo version 1 after correction by Canu version 1.6, and assembly version.4 by Wtdbg version 1.2.8 after correction with Canu version 1.6. Higher quality reads, which were corrected by Canu version 1.6 (-correct -p assembly useGrid = true corOutCoverage = 80 minReadLength = 5,000) representing above 80 × coverage, were used to generate assembly version.5 by SMARTdenovo and assembly version.6 by Wtdbg version 1.2.8. Taking all the assemblies into consideration, assemblies version.4 (380 Mbp) with the lowest contig number of 3,354 and the longest contig N50 of 571 kb, and version.5 (414 Mbp) with 2519 contigs with N50 of 483 kbp, were the best ones for further scaffolding and polishing. The two assemblies were merged by Quickmerge (Chakraborty et al., 2016). Finally, the assembly version 1 was produced after two rounds of polishing based on high-quality Illumina reads by Pilon v1.22 (Durand et al., 2016a; Supplementary Table 3).

### Scaffolding for Genome Assembly

Reads from the Hi-C library were preprocessed, with adapter sequences trimmed and low-quality bases removed. Quality-filtered reads were aligned to assembly version 1 using BWA version.7.17 (Li and Durbin, 2009). The resulting bam files together with the contigs from assembly version 1 were used as input for Juicer version 1.5 (Durand et al., 2016b). The contigs were clustered, ordered, and oriented using 3d-DNA (Dudchenko et al., 2017). Hi-C contact matrix based on neighboring interaction was visualized in Juicebox version 1.8.8 (Durand et al., 2016b). It identified 12 high-confidence clusters representing 12 pseudochromosomes (Supplementary Figure 3). Each pseudochromosome cluster was re-scaffolded by 3d-DNA before manual correction of contig dis-assembly, scaffold misjoins, and marginal adjustment. Finally, the chromosome framework was constructed along with the interspersed sequences. To fill and close gaps, PacBio reads were mapped to scaffolds using LR_Gapcloser,^2^ and the consensus sequences were polished for 5 rounds with Pilon. Redundancy was removed within interspersed reads by using Redundans (Pryszcz and Gabaldon, 2016). Contigs with lengths of less than 5 Kbp were removed. We combined the coverage depth distribution and the alignments using BlastN against the Nt database, to remove contaminations from other species. The contigs with low average coverage depth (<10×) or a high no-coverage ratio (>60%) were discarded, which might be caused by assembly errors or low base quality. The final assembly v1.1 was formed for the following analysis (Supplementary Table 3).

### Transcriptome Assembly and Analysis

A total of 262.55 million raw reads of RNA-seq in four tissues of Suanzao (Supplementary Table 1) were produced to assemble the transcripts, which would be used for gene modeling. RNA-seq data sets in fruits of Suanzao, Dongzao, Cuizao, Huping, Jinsixiaozao, Junzao, and Muzao, with > three replicates, were downloaded from the NCBI SRA database (Supplementary Table 13), and the typical RNA-seq analysis was conducted for identification of genes specifically expressed in Suanzao. The RNA-seq raw reads were assessed for quality control using FastQC (Brown et al., 2017) and trimmed by Trimmomatic version.33 (Bolger et al., 2014). Furthermore, processed reads were aligned to the genome assembly by using HiSat2 version 2.1 (Kim et al., 2015), and then the gene expression FPKM values were calculated by using StringTie version 1.3.3b (Pertea et al., 2016). The putative transcription factors (TFs) were predicted by PlantRegMap (Tian et al., 2020) with homology to *Arabidopsis thaliana*.

The *de novo* transcriptome assembly was conducted based on the high-quality reads using Trinity v2.0.6 (Grabherr et al., 2011), and the aligned reads against the genome assembly were subject to the second transcriptome assembly by using StringTie v1.3.3b. The third reference-genome-guided transcriptome assembly was constructed by using Trinity v2.0.6. The three assemblies were merged by CD-HIT v4.6 (Fu et al., 2012), and finally, a total of 85,445 unique transcripts were achieved.

### Repeat Identification and Gene Annotation

Both *de novo* and homology-based methods were used for repeat annotation. RECON v1.08 and RepeatScout version 1.0.5 (Price et al., 2005) integrated into RepeatModeler v1.0.10^3^ were employed to generate a *de novo* repeat library, which was then combined with RepBase (Bao et al., 2015) library to further characterize transposable elements (TEs). Other repeats were identified by RepeatMasker (version 4.0.7, rmblast-2.2.28) (Chen, 2004) based on homology-based methods. DupGen_finder^4^ was used to identify duplicated genes and classify them into five categories, i.e., Whole Genome Duplication (WGD), Tandem Duplication (TD), Proximal Duplication (PD), Transposed Duplication (TRD), and Dispersed Duplication (DSD) (Supplementary Table 7).

Putative protein-coding genes were identified based on the *ab initio*-, evidence-, and homology-based gene prediction methods. For *ab initio* gene prediction, coding sequences from *A. thaliana* and 1,440 single-copy orthologs from the Benchmarking Universal Single-Copy Orthologs (BUSCO) embryophyta odb9 database (Yang et al., 2011) were selected for parameter training in AUGUSTUS version 3.2.3 (Stanke et al., 2008). After five rounds of optimization, MAKER package version 2.31.9 (Cantarel et al., 2008) was used for the prediction of the gene model. For evidence-based gene prediction, 25309 transcripts from transcriptome assembly were aligned to the repeat-masked reference genome assembly with BlastN and TblastX from BLAST version 2.2.28 + (E-value cutoff of 10^–5^) (Boratyn et al., 2012), respectively. 76,620 protein sequences from *A. thaliana* (Swarbreck et al., 2008), *V. vinifera* (Jaillon et al., 2007), and *Z. jujuba* cv. Dongzao (Liu et al., 2014) were used as homology-protein evidence for gene annotation and were aligned to the genome assembly of Suanzao, which had TE and repeat masked by RepeatMasker version 4.0.7, by using BlastX. The alignments were manipulated with Exonerate v2.4.0 (Slater and Birney, 2005). Finally, gene model predictions of the three strategies were integrated based on the evidence by MAKER, and annotation edit distance (AED) was calculated to evaluate the performance of gene predictions. The completeness of gene annotation was assessed by BUSCO. All the predicted genes were searched against seven databases for functional annotation (Supplementary Table 5).

### Phylogenetic and Gene Family Analysis

Protein sequences of Junzao and Dongzao genomes (The NCBI accession number LPXJ00000000.2 for Junzao and JREP00000000.1 for Dongzao) and 21 additional species, i.e., *Ziziphus jujuba*, *Ochetophila trinervis*, *Morus notabilis*, *Parasponia andersonii, Trema orientale*, *Fragaria vesca*, *Prunus persica*, *Prunus mume*, *Prunus avium*, *Rosa chinensis*, *Rubus occidentalis*, *Pyrus bretschneideri*, *Malus domestica*, *Vitis vinifera*, *Populus trichocarpa*, *Medicago truncatula*, *Arabidopsis thaliana*, *Theobroma cacao*, *Coffea canephora*, *Daucus carota*, and *Oryza sativa*, were downloaded and used to identify orthologs by using Orthfinder2 (Seppey et al., 2019) (Supplementary Table 9). Orthologous genes were determined by all-versus-all BLASTP comparisons (Blast + v2.3.056) (Boratyn et al., 2012) with the E-value cutoff of 10^–5^. Multiple alignments were performed based on 149 orthologous single-copy concatenated protein-coding genes by using MAFFT v7 (Katoh and Standley, 2013), and the maximum likelihood (ML) phylogenetic tree was constructed by IQ-TREE v1.6.7 (Lam-Tung et al., 2015), with *O. sativa* as the outgroup and bootstrap test of 1,000 runs. This ML tree and the 1067 single-copy orthogroups, which are shared by a minimum of 87% of all the 23 assemblies, were then used as the inputs to estimate divergence time by the MCMCTree program in the PAML package v4.9h (Yang, 2007).

### Single Nucleotide Polymorphism Calling and Population Genetic Analyses

Resequencing data of 108 jujube accessions were downloaded from NCBI, for population genetic analysis (Supplementary Table 11). Clean reads were mapped to the Suanzao genome assembly using BWA version 7.17, and only reads with mapping scores > 30 were kept. For population genetic analysis, Freebayes was used for single nucleotide polymorphism (SNP) and genotype calling across all samples and 15,446,161 variable sites were identified out of 316,991,961 sites. The missing sites with genotype quality of <20 or depth of <3 were discarded, and a total of 7,471,374 SNPs were obtained. After filtering out the sites with MAF < 0.05 (Ghosh et al., 2018), 3,698,492 SNPs were retained for genotype imputation and phasing in BEAGLE (Browning and Browning, 2007).

Plink (Purcell et al., 2007) was used to filter out linkage sites with parameters (–file plink, –noweb, –make-bed, –allow-extra-chr), and 166,444 independent SNPs were identified for population structure estimation using ADMIXTURE (Alexander et al., 2009). A neighbor-joining tree was constructed using MEGA7 (Kumar et al., 2016) based on the distance matrix. Principal component analysis (PCA) was performed using GCTA (Yang et al., 2011). Linkage disequilibrium (LD) was calculated using PopLDdecay^5^ with parameters of “-MaxDist 300 -MAF 0.05 -Miss.2.”

The SMC + + (Terhorst et al., 2017) was employed to estimate the demographic history of the 35 selected jujube accessions, without phasing. With a generation time of 2 years and a mutation rate of 10^–8^, SMC + + results were used for the calculation of time periods and effective population size (*N*_*e*_).

### Synteny Analysis and Detection of Selection Signature

Based on the ortholog and paralog clusters identified by Orthofinder2 (Emms and Kelly, 2015), a collinear analysis was performed for the comparison of Dongzao-Suanzao, Dongzao-Junzao, Suanzao-Junzao using MCScan (Wang et al., 2012).

The allele frequency of each population was calculated in ANGSD (Korneliussen et al., 2014), and the composite likelihood ratio (CLR) was produced by using Sweepfinder2 (DeGiorgio et al., 2016) to scan the genome-wide sweeps based on a grid size of 2000 bp, which are the regions of significant deviation from the neutral site frequency spectrum (SFS).

## Results

### Genome Sequencing and Assembly

“Suanzao,” the sour jujube *Z. jujuba* Mill. var. *spinosa* from middle China, with significant morphological differences with “Dongzao” (Figures 1A–C), was selected for genome sequencing and the following assembly based on both Illumina short reads and PacBio long reads. Illumina sequencing generated ∼103.4 million paired-end 150-nt reads (∼16.487 Gbp, about 76 × coverage of the genome), with quality values of 97.14 and 93.96% reads for over 20 and 30, respectively (Supplementary Table 1). By k-mer analysis of short reads, the genome size was estimated to be ∼413 Mbp with a heterozygosity ratio of 1.21% and repeat frequency of 56.77%, and the error frequency was estimated to be 0.10% (Supplementary Figure 1 and Supplementary Table 2). And a total of 6,670,081 PacBio long reads were generated for genome assembly, i.e., 50.22 Gbp (approximately 120 × coverage of the genome), with an average read length of 7,529 bp (Supplementary Table 1 and Supplementary Figure 2).

PacBio reads were assembled to primary contigs based on the optimization of a combination of assembly software (see Methods for details of assembly procedures), which were polished with both PacBio long reads and Illumina short reads. As a result, a genome assembly Version v 1, comprised of 1918 contigs with N50 of 1.09 Mbp, was generated, with a total size of 409 Mbp (Supplementary Table 3). The Hi-C was employed for scaffolding into 12 pseudo-chromosomes (Supplementary Figure 3 and Figure 1D). The final assembly v1.1 consisted of 919 contigs and 540 scaffolds, and gene completeness reached up to 95.6% (Table 1 and Supplementary Table 3). The contig N50 is 2.1 Mbp, and the scaffold N50 is 30.3 Mbp. The longest contig is 11.3 Mbp, while the longest scaffold is 50.3 Mbp. The size of the assembled genome, 406 Mbp, is consistent with the genome size of 413 Mbp predicted by the k-mer analysis. 97% of the assembled genome could be covered by mapped Illumina short reads by BWA (Li and Durbin, 2009) or PacBio long reads by Minimap2 (Li, 2018). Compared with the published jujube genome assemblies of “Junzao” and “Dongzao” (Liu et al., 2014; Huang et al., 2016), the genome assembly of Suanzao has better continuity and completeness on length distribution of contigs and scaffolds (Supplementary Figure 4). It all showed the high quality and completeness of the Suanzao genome assembly.

**TABLE 1:** Comparison of three jujube genome assemblies.

	Junzao	Dongzao	Suanzao
Tissues for DNA extraction	Leaves of the mature tree	*in vito* culture tissues	Leaves newly branches on millennium-old root stock
Estimate of genome size (Mbp)	360	443	406
Chromosome number (2n)	2 × 12	2 × 12	2 × 12
Sequencing depth	227	249	120 (Pacbio) + 76 (Illumina)
Total length of scaffolds (bp)	351,115,537	437,645,007	406,163,984
Anchored scaffolds (Mbp)	293.7 (83.6%)	321.5 (73.6%)	380.3 (93.7%)
N50 length (scaffolds ≥ 100 bp)	754,884	301,045	30,278,369
N50 length (contigs ≥ 100 bp)	34,020	33,948	2,144,872
BUSCO genes	891 (93.2%)	851 (89.0%)	1376 (95.56%)
Gene number	27,443	31,067	25,089
Transposable elements (bp)	136,329,650 (38.8%)	204,918,483 (46.8%)	215,926,664 (53.16%)

### Genome Annotation

A total of 25,089 genes were predicted, and 91.15% of genes were annotated with AED < 0.5. The average lengths of gene regions, transcripts, coding sequences, and exons are 3,669 bp, 1485.14 bp, 1258.44 bp, and 258.68 bp, respectively (Supplementary Table 4). Moreover, 1181 transfer RNAs (tRNAs), 420 non-coding RNAs (ncRNAs), and 150 ribosomal RNAs (rRNAs) were identified (Supplementary Table 4). Based on sequence similarity, 23,283 (92.8%) of all predicted genes were assigned with functions against the protein-related databases (Supplementary Table 5). 1,376 (95.6%) out of 1,440 Embryophyta genes were identified in the “Suanzao” genome (Supplementary Table 6). Out of 1,376 genes, 1,320 genes (91.67%) were single-copy while 56 ones (3.89%) were duplicated.

We observed footprints of genes in the duplicate events, which happened in the different genomic evolutionary periods. Duplicated genes were classified into five categories, i.e., WGD, TD, PD, TRD, and DSD (Supplementary Table 7). Then Gene Ontology (GO) enrichment analysis was performed on duplicated genes. Five types of duplicates exhibited divergent functions. TRDs, TDs, and DSDs were enriched in the GO term of ATPase activity, and WGDs showed specific enriched GO terms which were not found in the other duplicates. Compared with the others, TD and PD shared more enriched GO terms related to nutrient reservoir activity, transferase activity, recognition of pollen, iron ion binding, polysaccharide binding, polysaccharide binding, oxidoreductase activity, and ADP binding.

Both *de novo* and homology-based methods were used for repeat identification. It was shown that more than half of the assembled genome (53.16%) were predicted to be TEs and/or repeats, of which 39.72% consisted of known TEs, 9.86% unknown, and 2.78% simple repeats. Most TE sequences were long terminal repeat-retrotransposons (LTR-RTs, 28.95%) with the dominant superfamilies of Gypsy (8.60%) and Copia (18.17%), and long interspersed nuclear element (LINE) and short interspersed nuclear element (SINE) accounted for 1.01 and 0.05%, respectively (Figure 1D and Supplementary Table 8).

### Comparative Genomics and Duplication Events

**FIGURE 2:**
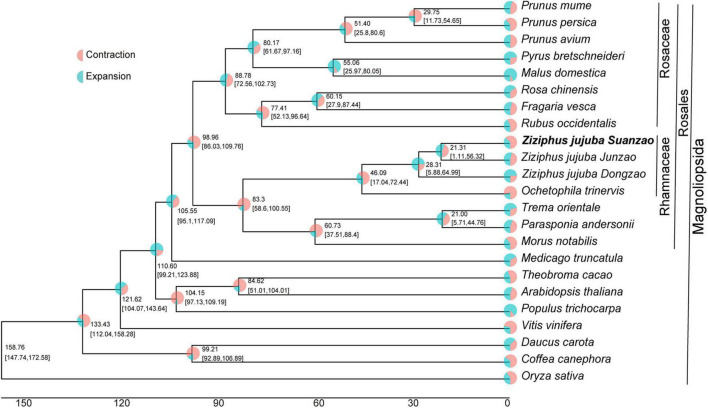
The phylogenetic tree of 23 genomes. Divergence time and proportions of gene families that underwent expansion or contraction are shown in the nodes. Bootstrapping supports (SH-aLRT/UFBoot) are presented along with the 95% CIs for each dating point in brackets.

To investigate the evolution of Suanzao, protein sequences of two jujube genome assemblies, and 20 additional Magnoliopsida species were selected for the comparative analyses (Supplementary Table 9). Based on protein sequence homology, 752,607 annotated genes from all 23 genomes were clustered into 627,068 orthogroups. Out of 627,068 orthogroups, 6750 orthogroups were shared and 149 orthogroups were single-copy (Supplementary Table 10). The phylogenetic tree was constructed based on 149 orthogroups of single-copy genes. And it identified the closest relationship of *Z. jujuba* to *Ochetophila trinervis* and their divergence time was 46.09 million years ago (Mya) in Rhamnaceae. The variant-formed time of *Z. jujuba Mill. var. spinosa* was estimated to be about 21.31 Mya being divergent from Junzao (Figure 2), and the divergence time with Dongzao was about 28.31 Mya. It is noted that Suanzao bore more gene contractions than those in the other jujube genomes. The results also suggested a speciation time of 83.3 Mya for Rhamnaceae clade and the neighbor clade (including *M*. *notabilis*, *P. andersonii*, and *T*. *orientale*), the divergence time of 98.96 Mya for Rhamnaceae and Rosaceae clades, and divergence time of 110.6 Mya for jujube and *A. thaliana*, which was in agreement with the previous analysis (Liu et al., 2014).

In plants, genome expansion and evolution are primarily driven by polyploidization caused by WGD events. We began by comparing the high synteny of one-to-one relationships among Dongzao, Junzao, and Suanzao with synteny and collinearity analyses. Analyses showed that these jujubes shared common whole-genome-duplication events in their evolutionary history (Supplementary Figure 5A). It was confirmed by the distribution curve at synonymous sites (Ks) calculated based on the orthologs in Suanzao v.s. Dongzao/Junzao, and Suanzao v.s. *Vitis vinifera* (Supplementary Figure 5B). Grape is often used for investigating the WGD events of eudicot genomes since its genome underwent minimal rearrangement following the γ event. Syntenic analysis of Suanzao and grape supported that Suanzao did not undergo recent genome duplication, and shared γ hexaploidization event with *V. vinifera* (Jaillon et al., 2007). However, a large number of chromosome rearrangements, especially inversions on chromosome #4, have been found in the Dongzao assembly, compared with the other two jujubes (Supplementary Figure 5A). In contrast, there was higher collinearity between Junzao and Suanzao, which is consistent with the results shown by the phylogenetic tree, and these genome evolution events suggested the recent split of Junzao and Suanzao following the split of Dongzao and Suanzao. Multiple inversions were observed in a comparison of Junzao and Suanzao on chromosomes #4, #10, and #6. The genome and chromosomes might be reconstructed for the most recent ancestor of flowering plants, referred to as the ancestral eudicot karyotype (AEK) (Badouin et al., 2017). The conserved chromosomes were identified in Suanzao assembly, for example, chromosome #7 was derived directly from AEK #3 (see red bars in Supplementary Figure 5C). Chromosomes #8 and #9 were derived from AEK5 (see orange bars in Supplementary Figure 5C), and chromosomes #5 and #10 were from AEK6 with isolated chromosomal rearrangement (see yellow bars in Supplementary Figure 5C).

### Population Structures in Jujube

**FIGURE 3:**
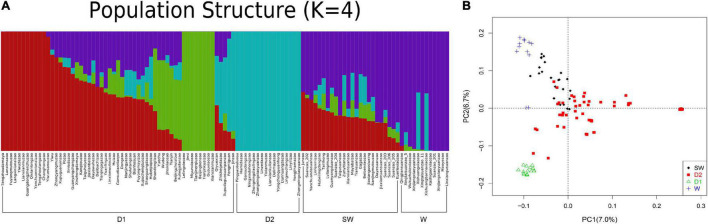
Populational structure of 109 *Ziziphus* accessions. **(A)** ADMIXTURE plot of 109 *Ziziphus* accessions shows four subpopulations. **(B)** Principal component analysis (PCA) results for four subpopulations. “Linxiangusuanzao” in the wild subgroup is the genome assembly in this study.

To unveil the ancient genetics components of Suanzao and the genetics structure and relationships between Suanzao and other jujube accessions, 108 accessions downloaded from NCBI were divided into two genetic components (domesticated and wild groups) with K = 2, and if K = 4, the wild group was further divided into two subgroups of W for wild, SW for semi-wild, and two domesticated ones of D1 and D2, which was consistent with their geographical distributions (D1 mainly in West China and D2 in East China and Southeast China) (Figure 3 and Supplementary Table 11). PCA results revealed four major clusters, which were consistent with the ADMIXTURE result of D1, D2, semi-wild and wild sub-groups. “Linxiangusuanzao” is the genome assembly in this study, which is placed in the wild subgroup, while Dongzao is in D2 (Figure 3).

SMC + + was employed on unphased genomes to infer the changes of effective population size (*N*_*e*_) over the historical time in jujube (Terhorst et al., 2017). The results suggested that the wild and domesticated jujube were divergent ∼1,000 kya (thousand years ago) to 6 kya with remarkably similar variation sites and experienced a dramatic contraction of *N*_*e*_, under the influence of the climate change in the Last Glacial Period (∼70 to 1.5 kya) with the nadir at ∼5.5 kya (Supplementary Figure 6). Meanwhile, wild jujubes seem to have experienced a population bottleneck ∼400 to 230 kya before the onset of the decline of *N*_*e*_ (the green line in Supplementary Figure 6), while some domesticated populations underwent a population bottleneck event between ∼6 kya and 3.5 kya (the red and yellow lines in Supplementary Figure 6), which might be related to the selection of human society. In particular, the population size of *Z. mauritiana* Lam. (Maoyezao) declined sharply ∼400 kya, followed by a bottleneck until ∼230 kya (the black line in Supplementary Figure 6).

### Detection of Selection Signature

Linkage disequilibrium (LD) decayed to r^2^ = 0.2 at 20 Kbp among three groups. Surprisingly, the slope of the LD-decay curve dropped the fastest for the wild, which suggested the hypothesis of selective sweep (Supplementary Figure 7). Selection history in West China resulted in a slower reduction of LD decay in the D1 subgroup compared with the D2 subgroup in East China. It was reported that the wild group achieved a faster decline of LD decay (Guo et al., 2020).

**FIGURE 4:**
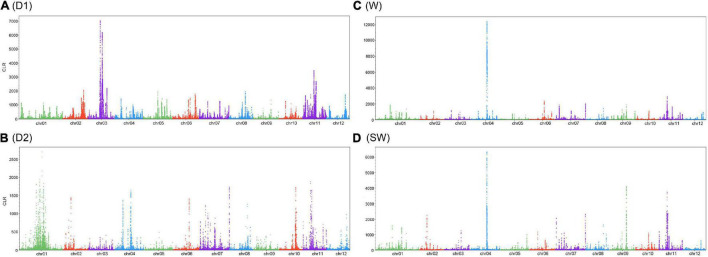
Selection signals with a calculation of composite likelihood ratio (CLR) in three sub-groups. **(A)** D1; **(B)** D2; **(C)** wild; **(D)** semi-wild.

Genomic regions were under selection signals with a calculation of CLR. In total, CLR identified putative regions which covered 395 genes on chromosomes in the D1 subgroup, 396 genes in the D2 subgroup, 421 genes in the wild group, and 415 genes in the semi-wild group (Figure 4 and Supplementary Table 12), while the specific selections were found in the three groups, for example, sharp peaks on chromosome #3 in the D1 subgroup, #1 in the D2 subgroup, #4 in wild subgroup and #4 in the semi-wild subgroup. Interestingly, some of these genes are related to environmental adaptation. For example, the gene of Zijuj10G0113500, located on chromosome #10, was under the distinct peaks in all three groups, with the biggest values of CLR in D1 and D2 subgroups, and this gene is involved in the biological processes related to responses to salt stress, water deprivation, cold, abscisic acid, sucrose stimulus, phosphate starvation, and toxic substance. Two genes, Zijuj03G0095400 and Zijuj03G0096000, within the peak of chromosome #3 in the D1 subgroup were annotated as regulation in glycerol catabolic process and metal ion binding in the development of seed and root. Gene Zijuj01G0154900 within the peak of chromosome #1 in the D2 subgroup is involved in 1,3-beta-D-glucan synthase complex in a response to sucrose, salicylic acid-mediated signaling pathway. Genes, Zijuj02G0076100 and Zijuj02G0076200, in the wild subgroup within the peak of chromosome #2 are involved in a response to salt stress, abscisic acid-activated signaling pathway, and defense response (Supplementary Table 12).

### Expression of Genes Under Selection in Fruits

**FIGURE 5:**
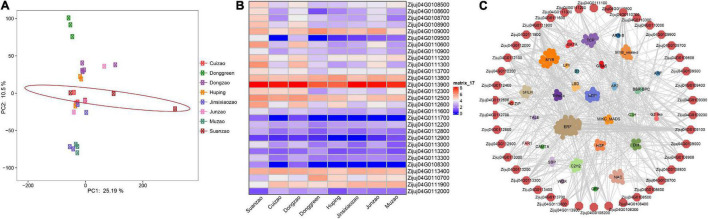
Expressions of genes in fruits of jujube accessions. **(A)** PCA results of 8 jujube accessions based on gene expressions in fruits. **(B)** Heatmap of expressions of genes in fruits, which are under strong selection on chromosome 4 in the wild. **(C)** Networks of transcription factors which are predicted for the genes under strong selection on chromosome 4 in the wild, based on PlantRegMap database.

To test the expression genes under selection in fruits, we downloaded the RNA-seq data of other accessions, including Suanzao, Dongzao, Cuizao, Huping, Jinsixiaozao, Junzao, and Muzao, from the NCBI SRA database (Supplementary Table 13), and conducted the typical RNA-seq expression analysis. The results showed that Suanzao fruits were of larger expression variations in PCA analysis, and different from the other jujubes (Figure 5A). The differentially expressed genes (DEGs) in Suanzao varied from 683 ones against Junzao to 4535 ones against Donggreen. The DEGs between Suanzao and the others were overlayed with the 53 genes (chr04: 17526000-19526000), which are under the highest peak of selection signature on chr04 in semi-wild and wild subgroups (Figure 4C and Supplementary Table 12), and then we found that there were two groups of genes, including 25 up-regulated and 4 down-regulated ones in Suanzao, compared with the other jujube accessions (Figure 5B). These results suggested the 53 genes within this selection region could be involved in the development and maturation of fruit tissue in Suanzao, of which 42 genes were predicted as 182 transcription factors (TFs) based on the PlantRegMap database. Furthermore, we revealed a network that showed the links between these 42 genes and their predicted 182 TFs. The 182 TFs were enriched in 30 families, which appeared as potential direct regulators of the downstream-regulated genes, while ERF family members were most numerous (Figure 5C).

## Discussion

With the Suanzao genome assembly, we identified large inversions on chromosome 4 between Suanzao/Junzao and Dongzao, albeit of high collinear blocks among the three genome assemblies. In fact, in contrast to the close relatives of the jujube in the Rosaceae, fewer chromosome fissions, fusions, and rearrangements occurred in the jujube genome, compared with the peach and apple genomes (Huang et al., 2016).

### Genome Selection and Population Evolution

The elucidation of the Suanzao genome has also provided unique insights into the population evolution and selection of jujube plants. Cultivated jujubes were domesticated from their wild ancestors, and experienced the artificial selection into two subgroups of D1 and D2, which were consistent with their geographical distributions and the previous report on the subgroups I and II (Huang et al., 2016). We further explored their population history and found the significant differences among the sub-groups of wild, semi-wild, D1, and D2 accessions (Supplementary Figure 11 and Figure 3), and D1 and D2 matched the geographical distribution of West China and East/Southeast China, respectively, which suggested the independent domestication history. It was also reported that peach (*Prunus persica*) accessions were grouped according to their geographical origin of east and west (Verde et al., 2013), and the wild plants are most closely related to the peach of northern China ecotypes (Yoon et al., 2006). The bottleneck of peach is reflected in the decrease of nucleotide diversity observed when moving from eastern to western varieties, and in comparison with the eastern varieties and wild relatives, the western varieties showed a relatively slow LD decay (Verde et al., 2013). Interestingly, the jujube showed similar results in this study. Geographical isolation is widely discovered in fruit trees. Pear (*Pyrus*) originated in southwestern China domestication went toward the two directions of both east and west, and then Asian and European pears were formed separately (Wu et al., 2018). Apricot (*Prunus armeniaca*), which had long been considered to have originated from China, form two different gene pools of Chinese and European apricots, but now the European cultivated apricots were found to originate from the Northern Central Asian wild population, while the Chinese cultivars originated from Southern Central Asian (Groppi et al., 2021).

The chromosomes of 2, 3 (highest peak), and 11 are all of the strong selection signals in the D1 subgroup, and the genes are valuable to be explored for their functions. For example, a whole-genome association study was conducted and the causal gene of ZjFS3 as ethylene-responsive transcription factor on chromosome #3 associated with fruit shape and kernel shape (Guo et al., 2020). Meanwhile, in the chromosomes of 1, 4, and 10 in the D2 subgroup, 4 in the wild and 4 in the semi-wild were with strong selection signals in selective sweep results and might be potentially related to artificial selection and natural adaption, respectively. This exploration revealed the various consequences of artificial selection during jujube domestication and elucidated the history of jujube domestication.

### Genes Under Selection for Stress Resistance and Fruit

We found a strong selection of genes in three subgroups, which might be associated with stress response in jujube. For example, the gene of Zijuj10G0113500, located on chromosome #10, was under the distinct peaks in all three groups and involved in the biological processes related to responses to stresses. We also searched the genomic regions with the highest peak of selection signals on chromosomes #4 in wild, for the genes playing roles in the bioactive ingredients of jujube fruits and seeds. Flavonoid and jujuboside could be the potential bioactive components showing beneficial antioxidative effects and tastes in jujube seeds (Seo et al., 2013; Chen et al., 2017). The highest peak on chromosome #10 in the wild did not cover the common 45 genes shared by the three subgroups, and after examining this peak, we found two key genes (Zijuj10G0134000 and Zijuj10G0134100) which are under unique selection in wild and annotated as dammarenediol 12-hydroxylase and catalyze the hydroxylation of dammarenediol-II to yield protopanaxadiol in ginsenoside biosynthesis, a class of tetracyclic triterpenoid saponins (Lu et al., 2018).

Jujuboside A (JuA) is one of the main components of saponin in SZS, i.e., jujube dried seeds (Otsuka et al., 1978). Our results showed that JuA contents ranged from.034 to.093%, with an average of.059% in 61 Suanzao cultivars, while jujuboside B (JuB) was ranged from.005 to.036%. This finding suggested the importance of saponins and their evolution selections in SZS. The gene Zijuj10G0136000 annotated as fatty acyl-ACP thioesterase B neighboring to the above two genes on chromosome #10 might play an essential role in *de novo* fatty acid synthesis, and potentially influence the flavor of the jujube fruits and seeds. Notably, we found that the expression of gene Zijuj10G0108400 in Suanzao was significantly up-regulated than those in others. The gene Zijuj10G0108400 is annotated as 6-phosphogluconate dehydrogenase (6PGDH) which is involved in the oxidative phase of the NADP-malic enzyme (NADP-ME). It was reported that during fruit ripening of sweet pepper (*Capsicum annuum* L.), both 6PGDH and NADP-ME activity both increased and are key to maintaining the supply of NADPH which is required for different NADPH-dependent processes (Munoz-Vargas et al., 2020). For example, proline biosynthesis requires NADPH and its content increased during fruit ripening, which is related to different plant stresses (Bouthour et al., 2015; Gonzalez-Gordo et al., 2019). NADP-ME is closely associated with the production of malate, which contributes directly to the acid taste of the fruit (Yao et al., 2009). In addition, the gene Zijuj10G0113400 in Suanzao whose fruit size is small (Figure 1C), is annotated as zinc finger protein and showed a higher expression than those in others. It was reported that a zinc finger protein negatively regulates pericarp cell size to control fruit size in tomatoes (Zhao et al., 2021), and similarly, it is reasonable to suspect that zinc finger protein (Zijuj10G0113400) might be involved in fruit size control in Suanzao. The Glutathione peroxidases (GPXs) genes were shown to have enhanced oxidative stress tolerance of the peach fruit in the late stage of ripening (i.e., the starting of senescence), and up-regulated expression of GPXs dramatically delayed the ripening of postharvest peach fruit (Huan et al., 2016). Interestingly, the expression of the GPX gene (Zijuj04G0110700) in Suanzao was significantly decreased (Figure 5B), which suggested its potential involvedness in fruit ripening. It was reported that the jujube fruits of the wild accessions softened more easily than those of the cultivated species (Guo et al., 2020), and therefore, gene Zijuj04G0110700 might contribute to the significantly extended postharvest shelf life of fleshy fruits in cultivated jujubes compared with wild groups.

These genes are interesting under the selection in the wild and valuable for further function confirmation in the future, for example, creating mutants and screening for the candidate genes just like those in maize (Wu et al., 2018). This study provided a valuable genomic resource for jujube improvement, especially the genes under significant selection.

## Data Availability Statement

This Whole Genome Shotgun project has been deposited into DDBJ/ENA/GenBank (Accession number: JAEACU000000000). The raw sequence data of whole genome sequencing and RNA sequencing in *Ziziphus jujuba* Mill. var. *spinosa* have been deposited into NCBI Bio-Project (Accession number: PRJNA542987). The final assembly is available with the accession number JAEACU010000000.

## Author Contributions

L-YS, Y-MM, J-FM, and S-GJ conceived the study. HLu, X-MW, S-GJ, HLi, S-SZ, K-HJ, SN, Y-TB, R-GZ, Q-ZY, Y-HC, J-YL, YL, S-WZ, and J-FM performed the sequencing and bioinformatics analysis. L-YS, X-LW, S-SZ, and X-JQ collected the samples and extracted the DNA. S-GJ, X-MW, and J-FM wrote the manuscript. S-GJ and X-MW edited the manuscript. All authors read and approved the final manuscript.

## Conflict of Interest

R-GZ, Q-ZY, and Y-HC were employed by the company Beijing Ori-Gene Science and Technology Co., Ltd. S-WZ was employed by the company Hebei Hemuliyuan Agricultural Science and Technology Co., Ltd. The remaining authors declare that the research was conducted in the absence of any commercial or financial relationships that could be construed as a potential conflict of interest.

## Publisher’s Note

All claims expressed in this article are solely those of the authors and do not necessarily represent those of their affiliated organizations, or those of the publisher, the editors and the reviewers. Any product that may be evaluated in this article, or claim that may be made by its manufacturer, is not guaranteed or endorsed by the publisher.

## Footnotes

1
https://github.com/ruanjue/wtdbg

2
https://github.com/CAFS-bioinformatics/LR_Gapcloser

3
http://www.repeatmasker.org/RepeatModeler

4
https://github.com/qiao-xin/DupGen_finder

5
https://github.com/BGI-shenzhen/PopLDdecay
