# Supplementary material for: Chromosome-Scale Genome Assembly for Chinese Sour Jujube and Insights Into Its Genome Evolution and Domestication Signature
Source: Front Plant Sci. 2021 Nov 24;12:773090. doi: 10.3389/fpls.2021.773090 (PMC8652243; doi:10.3389/fpls.2021.773090)
Supplement: Supplementary file 1 [file Data_Sheet_1.docx]

Supplementary Material

## Supplementary Figures


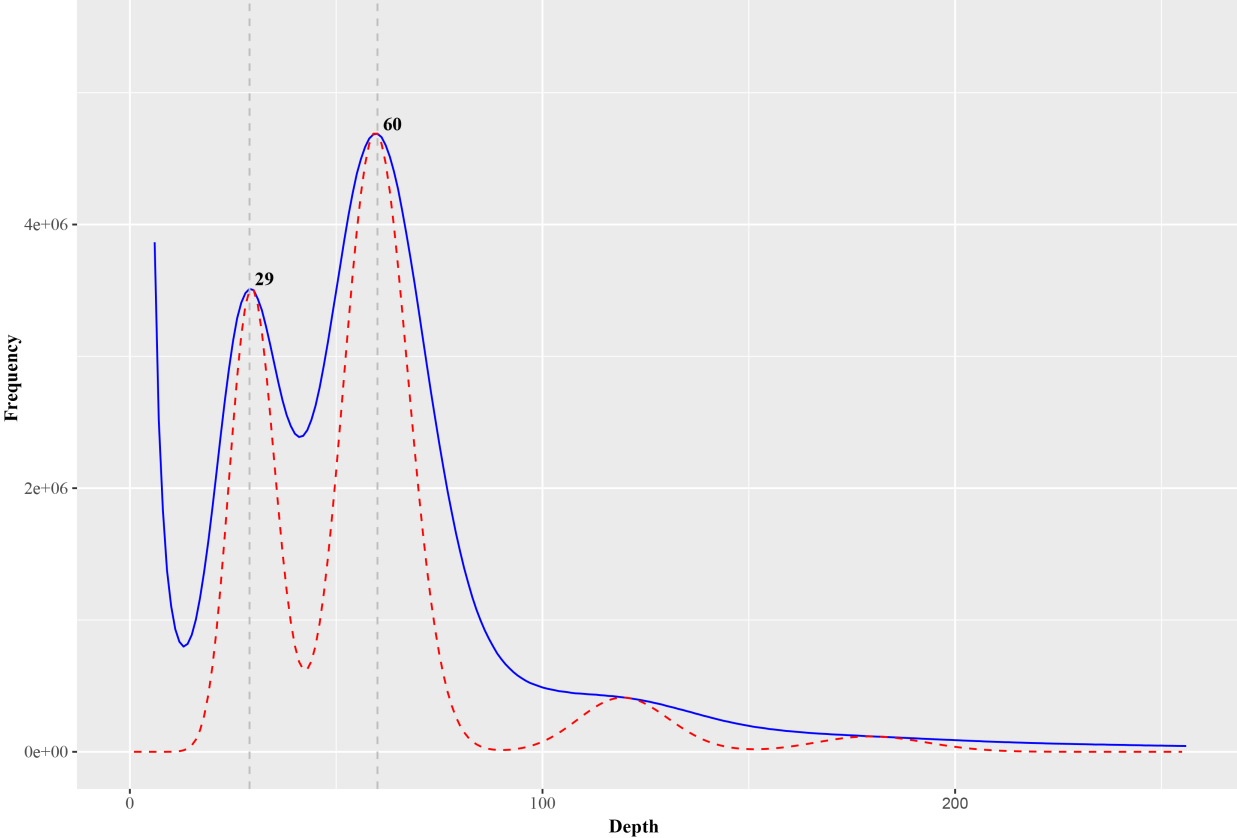


**Supplementary Figure 1.** K-mer frequency distribution at k-mer size of 17. K-mer refers to an artificial sequence division of K nucleotides. From k-mer frequency, genomic characteristics (genome size, repeat structure and heterozygous rate) could be estimated. Peaks at depths of 29 and 60 were noted, and the red dash line denotes the simulation based on the Poisson model.


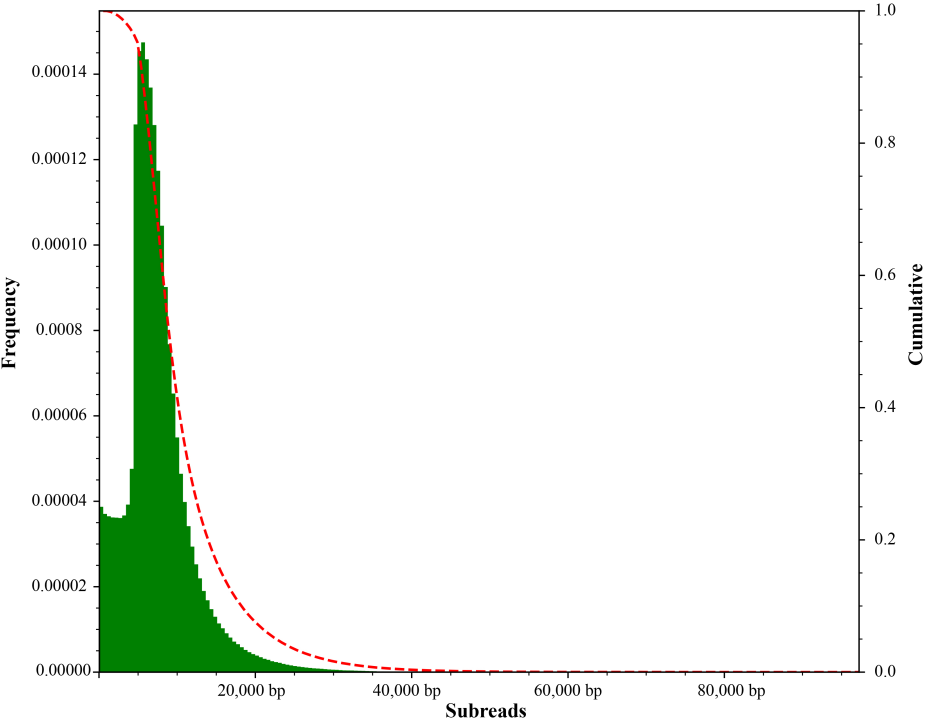


**Supplementary Figure 2.** Length distribution of PacBio subreads. The frequencies of subread length were plotted in green bars, and the cumulative ratio was shown in red dash lines.


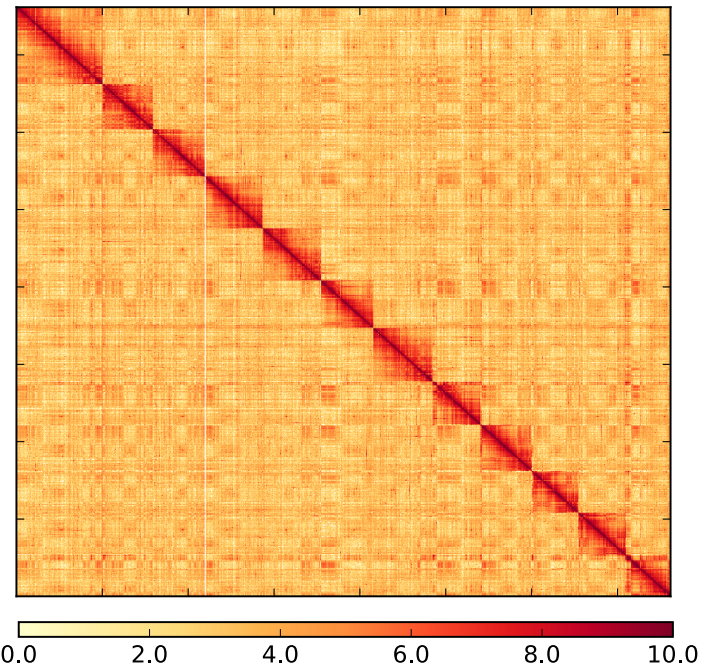


**Supplementary Figure 3.** The Hi-C assisted assembly of *Z. jujuba* Mill. var. *spinosa*.


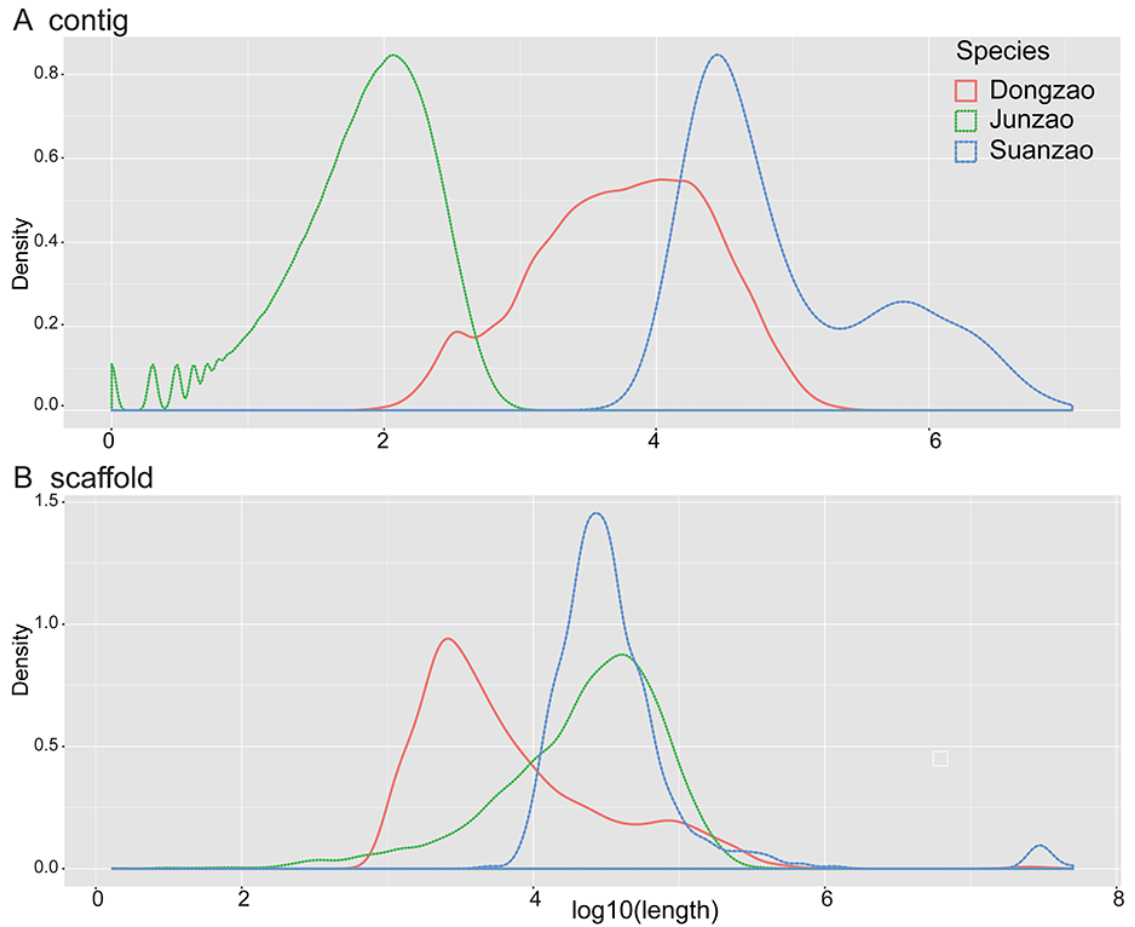


**Supplementary Figure 4.** Length distributions of contigs and scaffolds for Suanzao, Dongzao, and Junzao genomes. Length distribution of contigs (A) and scaffolds (B) are shown from three assemblies, red for Donzao, green for Junzao, and blue for Suanzao.


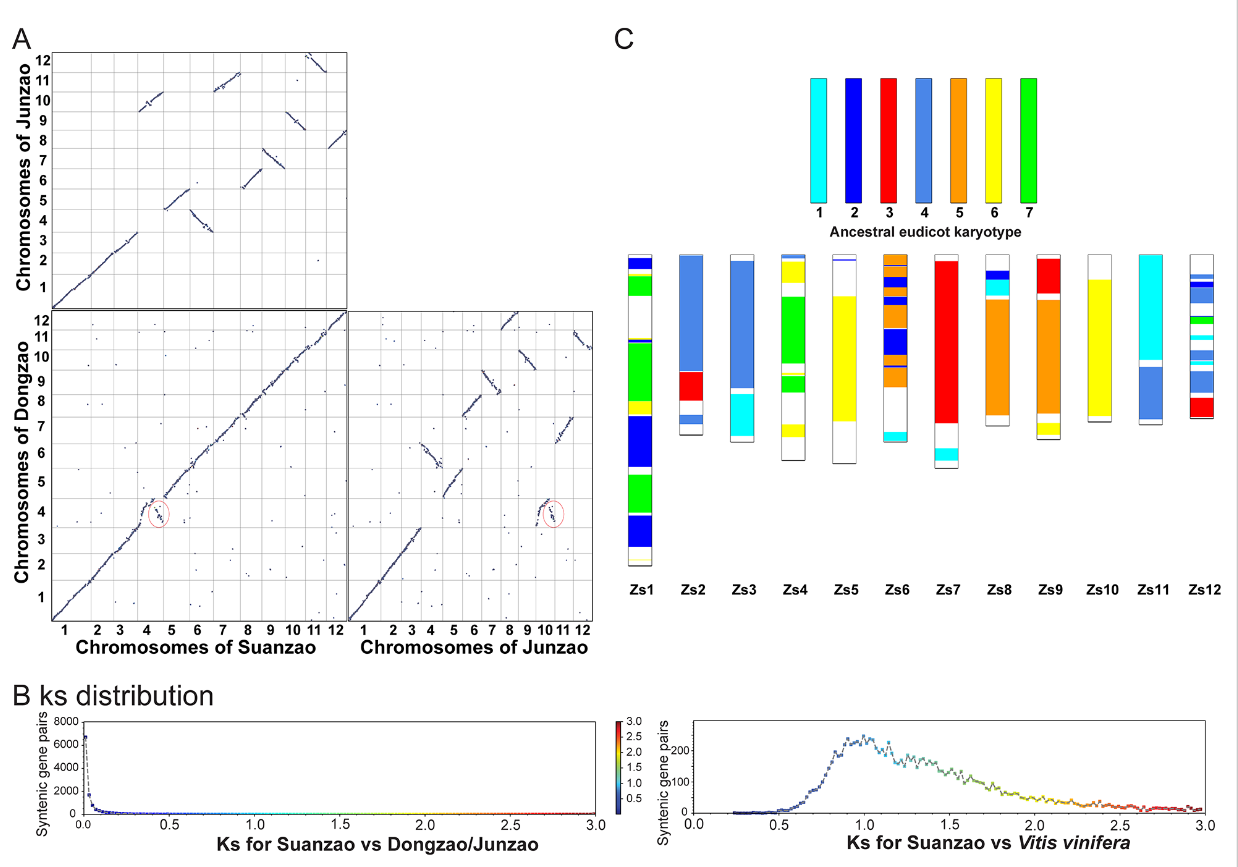


**Supplementary Figure 5.** Evolutionary analyses of the Suanzao genome. A. Collinearity analysis among the three jujube genomes for 12 chromosomes. B. Ks distribution in Suanzao v.s. Dongzao/Junzao (left) and Suanzao v.s. Vitis vinifera (right). C. Evolutionary scenario of the Suanzao genome from the ancestral eudicot karyotype (AEK) illustrated with seven colors (top). The Suanzao genome is illustrated at the bottom with different colors for 12 chromosomes reflecting the origin from the seven ancestral chromosomes from AEK.


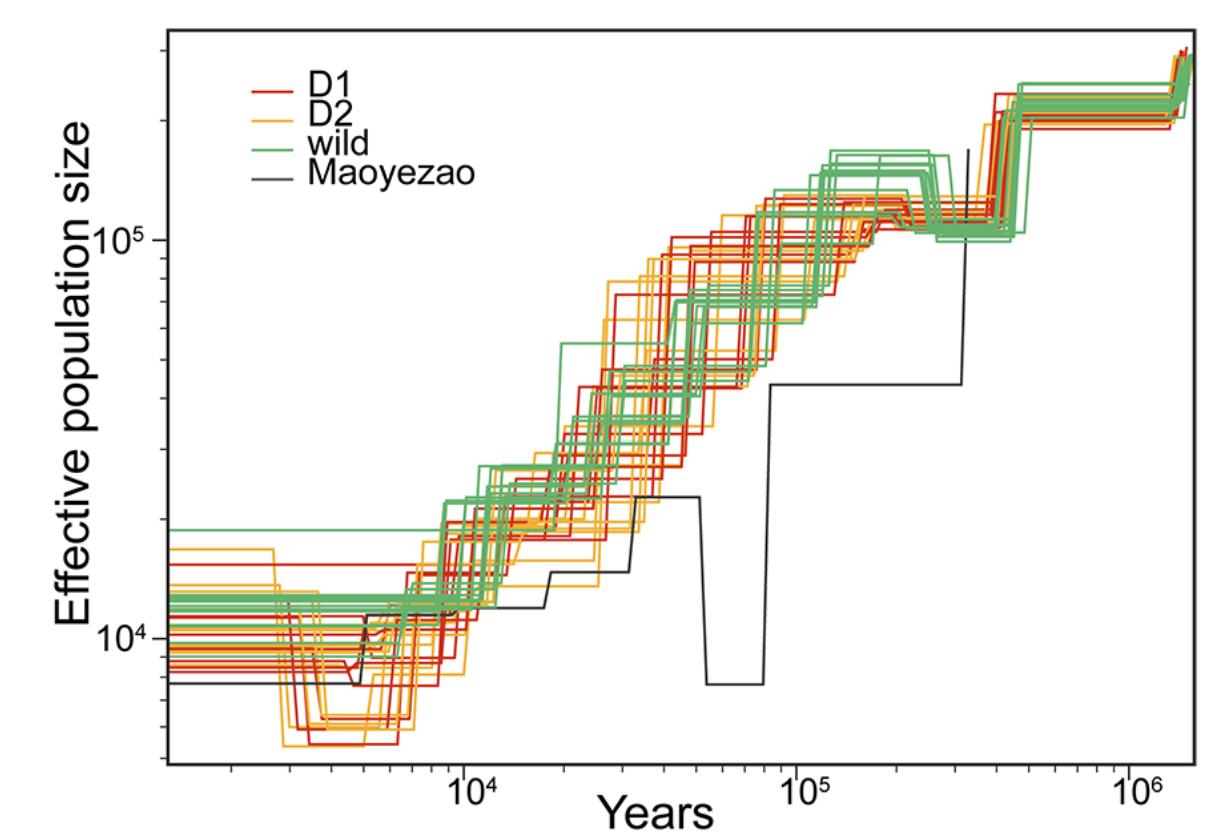


**Supplementary Figure 6.** Population evolutionary history of D1, D2, and wild subgroups, together with Maoyezao.


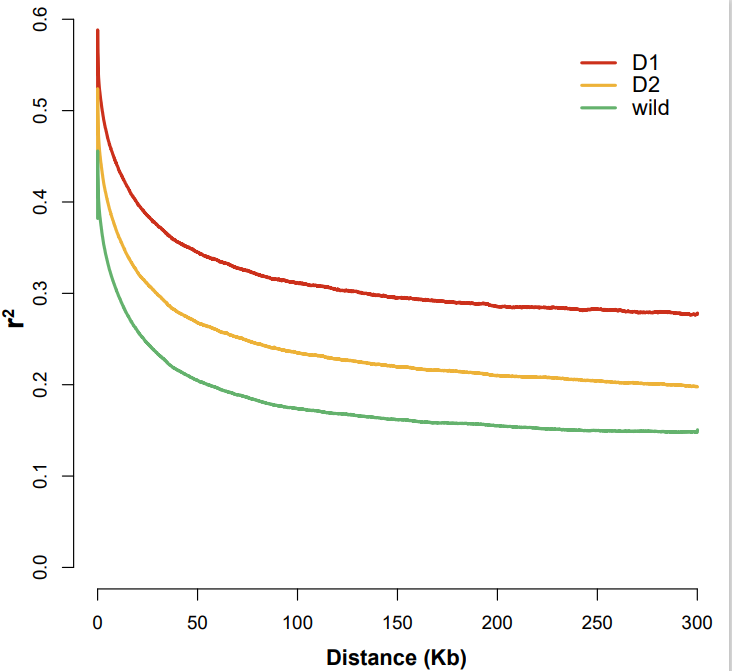


**Supplementary Figure 7.** Linkage disequilibrium (LD) decay of the three subgroups.
